# Principal component analysis of adipose tissue gene expression of lipogenic and adipogenic factors in obesity

**DOI:** 10.1186/s12902-023-01347-w

**Published:** 2023-04-27

**Authors:** Naghmeh Jannat Ali Pour, Hossein Zabihi-Mahmoudabadi, Reyhane Ebrahimi, Mir Saeed Yekaninejad, Seyyed Mohammad Reza Hashemnia, Reza Meshkani, Solaleh Emamgholipour

**Affiliations:** 1 Department of Clinical Biochemistry, School of Medicine, Tehran University of Medical Sciences, Tehran, Iran; 2 Department of Surgery, School of Medicine, Sina Hospital, Tehran University of Medical Sciences, Tehran, Iran; 3 Department of Neurogenetics, Max Planck Institute for Multidisciplinary Sciences, Göttingen, Germany; 4 Department of Epidemiology and Biostatistics, School of Public Health, Tehran University of Medical Sciences, Tehran, Iran

**Keywords:** Adipose tissue, Obesity, Lipogenesis, Adipogenesis

## Abstract

**Objective:**

A better understanding of mechanisms regulating lipogenesis and adipogenesis is needed to overcome the obesity pandemic. We aimed to study the relationship of the transcript levels of peroxisome proliferator activator receptor γ (PPARγ), CCAAT/enhancer-binding protein alpha (C/EBP-α), liver X receptor (LXR), sterol regulatory element-binding protein-1c (SREBP-1c), fatty acid synthase (FAS), and acetyl-CoA carboxylase (ACC) in subcutaneous adipose tissue (SAT) and visceral adipose tissue (VAT) from obese and normal-weight women with a variety of anthropometric indices, metabolic and biochemical parameters, and insulin resistance.

**Methods:**

Real‐time PCR was done to evaluate the transcript levels of the above‐mentioned genes in VAT and SAT from all participants.

**Results:**

Using principal component analysis (PCA) results, two significant principal components were identified for adipogenic and lipogenic genes in SAT (SPC1 and SPC2) and VAT (VPC1 and VPC2). SPC1 was characterized by relatively high transcript levels of SREBP1c, PPARγ, FAS, and ACC. However, the second pattern (SPC2) was associated with C/EBPα and LXR α mRNA expression. VPC1 was characterized by transcript levels of SREBP1c, FAS, and ACC. However, the VPC2 was characterized by transcript levels of C/EBPα, LXR α, and PPARγ. Pearson’s correlation analysis showed that unlike SPC2, which disclosed an inverse correlation with body mass index, waist and hip circumference, waist to height ratio, visceral adiposity index, HOMA-IR, conicity index, lipid accumulation product, and weight-adjusted waist index, the VPC1 was positively correlated with above-mentioned obesity indices.

**Conclusion:**

This study provided valuable data on multiple patterns for adipogenic and lipogenic genes in adipose tissues in association with a variety of anthropometric indices in obese subjects predicting adipose tissue dysfunction and lipid accumulation.

**Supplementary Information:**

The online version contains supplementary material available at 10.1186/s12902-023-01347-w.

## Introduction

Obesity is believed to be a principal factor for the development of insulin resistance, type 2 diabetes, non-alcoholic fatty liver, hypertension, and cardiovascular disorders. Over recent decades, the prevalence of obesity has substantially risen worldwide. In Iran, the prevalence of obesity has increased to about 26% in 2020 and based on a very recent study, the rate of obesity and overweight was reported to be higher among Iranian women than men [1–3]. Although white adipose tissue has been considered an active organ secreting several hormones and adipocytokines controlling lipid and glucose metabolism, it is still the principal contributor to the energy reservoir [4].

Adipose tissue dysfunction is a principal contributor to obesity and related metabolic abnormalities which primarily result from impaired lipogenesis and adipogenesis [5–7]. At the molecular level, adipogenesis and lipogenesis are regulated by the coordinated and highly complex transcriptional network comprising of peroxisome proliferator activator receptor γ (PPARγ), CCAAT/enhancer-binding protein alpha (C/EBP-α), liver X receptor (LXR), and sterol regulatory element-binding protein 1c (SREBP1c) [8–10]. PPARγ, a member of the nuclear-receptor superfamily, has been identified as the master regulator of adipogenesis as well as the main regulator of energy homeostasis in adipose tissue. C/EBPα is a family member of transcription factors. C/EBPs contribute to the regulation of adipocyte differentiation in cooperation with PPARγ and LXRα, a transcription factor belonging to the nuclear receptor superfamily which is involved in a wide range of metabolic pathways, including energy metabolism, inflammation, and particularly adipogenesis and lipogenesis [11–13].

Although the results are highly controversial, data from clinical studies indicated that the dysregulation of the aforementioned molecules is linked to obesity and associated metabolic abnormalities, including insulin resistance [14–16]. For instance, some studies demonstrated a higher expression of PPARγ and SREBP1c in obese subjects, while others have observed the opposite results in this regard. Moreover, a limited number of data showed a dysregulated expression of LXRα in the context of obesity in humans [14].

Today, a piece of considerable evidence indicated an alteration in the gene expression of the main lipogenic enzymes; fatty acid synthase (FAS) and acetyl-CoA carboxylase (ACC) which are associated with adipocyte lipid accumulation in obesity, although with contradictory findings [10, 17, 18]. For instance, Ranganathan G et al*.* reported the down-regulation of FAS in adipose tissues of subjects with impaired glucose tolerance [19]. In contrast, another study reported the up-regulation of FAS in adipose tissue of obese subjects which was linked to impaired insulin sensitivity and visceral fat accumulation [20].

Despite the considerable, but conflicting data on the role of dysregulated expression of lipogenic and adipogenic factors in the context of obesity in humans, there is still boundless enthusiasm in the search for this area. It is crucial to mention that the white adipose tissue is mainly distributed in two discrete anatomical depots; subcutaneous adipose tissue (SAT) and visceral adipose tissue (VAT). Remarkably, SAT and VAT are markedly distinct from each other in cellular, molecular, physiologic, clinical, and prognostic characteristics [21, 22].ence, the study of adipose tissue is principal to the understanding of metabolic abnormalities pertinent to the initiation and development of obesity. Moreover, there is still a great deal of work that must be done to unravel the precise role of adipogenic and lipogenic factors in the concept of obesity in humans.

Nowadays, a variety of anthropometric indices have been proposed to monitor and predict metabolic complications in obesity. These include body adiposity index (BAI), The abdominal volume index (AVI), visceral adiposity index (VAI), weight-adjusted waist index (WWI), conicity index (CI), waist-to-hip ratio (WHR), waist circumference (WC), and waist-to-height ratio (WHtR) [23, 24]. Thus far no study has assessed the association of lipogenic and adipogenic factors with a variety of anthropometric indices in obese subjects.

Besides, the regulation of adipose tissue expansion by targeting adipogenesis and adipogenesis has emerged as a possible therapeutic approach to obesity-associated metabolic complications. Therefore, a better understanding of mechanisms regulating lipogenesis and adipogenesis and complicated cross-talk between them is needed to develop appropriate therapeutic strategies to obesity. Noteworthy, there is a sexually dimorphic pattern of adipogenic and lipogenic genes [25, 26]***.*** Moreover, despite increasing concern to women’s health, further studies are still needed to improve their health since obesity is more prevalent in women than men in most countries. This issue highlights an urgent need to develop relevant sex-based therapeutic avenues for obesity-related disorders and obesity. Hence, the current study was conducted to simultaneously measure the mRNA expression of PPARγ, C/EBPα, LXR, SREBP1c, FAS, and ACC in SAT and VAT from obese women with obesity and those with normal-weight. Moreover, this study aimed to evaluate the relationship of the aforementioned genes with a variety of anthropometric indices, metabolic and biochemical parameters, and insulin resistance as well as with each other.

## Methods

### Obese and normal-weight groups

This case–control study protocol was approved by the ethics committee of Tehran University of Medical Sciences (IR.TUMS.Medicine.REC.1397.702) in compliance with the principles of the Declaration of Helsinki. All methods were performed in accordance with the relevant guidelines and regulations. Written informed consent was obtained from each individual before participation.

A total of 46 subjects (age range 20–53 years) were studied, including 24 obese women (body mass index; BMI ≥ 30 kg/m^2^) who underwent bariatric surgery and 22 normal-weight women (BMI ≤ 25 kg/m^2^) who underwent elective surgeries, including inguinal hernia or cholecystectomy. The age range was between 20 and 53 years for normal weight women and between 20 and 48 years for women with obesity. The obese group and normal-weight group were recruited from the Bariatric Surgery Center at Erfan Hospital and from the center of advanced laparoscopic surgeries at Sina and Loqman Hakim hospitals, respectively. All participants were of Iranian ethnicity.

The exclusion criteria for obese and non-obese groups were as follows: 1) had cardiovascular diseases, diabetes, malignancy, acute or chronic inflammatory or infectious diseases, known renal and liver dysfunction; 2) treated with medications for weight loss during the previous 6 months; 3) was currently pregnant or lactating; 4) was menopause; 5) had a surgery history during the previous 6 months, and 6) was currently smoking.

## Biochemical and laboratory measurements

The peripheral blood sample was drawn from the antecubital vein by a sterile venipuncture in the morning after an overnight fast before the start of the surgery. Fasting blood samples were centrifuged at 1200 × g for 10 min at 4 °C, and serum was collected and used for measuring fasting blood glucose (FBG), high-density lipoprotein cholesterol (HDL-C), low-density lipoprotein cholesterol (LDL-C), triglyceride (TG), total cholesterol (TC), alkaline phosphatase (ALP), aspartate aminotransferase (AST), alanine aminotransferase (ALT), uric acid, urea high sensitivity-reactive protein (hs-CRP), and insulin. The insulin resistance was calculated using the homeostasis model assessment of insulin resistance (HOMA-IR) formula: FBG (mg/dL) × fasting blood insulin (µU/mL) / 405. Resting blood pressure was measured three times on the right arm using a manual sphygmomanometer in seated participants.

## Assessment of adiposity indices

BMI was calculated as weight in kg divided by the square of height in meters. In detail, WC was measured midway between the inferior angle of the ribs and the suprailiac crest at the end of a normal expiration. Hip circumference was measured at the maximum circumference over the buttocks using non-stretchable tape. WHR was calculated based on the ratio of WC in cm divided by hip circumference in cm. WHtR was assessed as WC in cm divided by height in cm. WHR and WHtR were also calculated as WC in cm divided by hip circumference in cm and WC in cm divided by height in cm, respectively.

BAI, another index of obesity, was calculated using the following formula:

$$=\frac{hip circumference \left(cm\right)}{{height \left(m\right)}^{1.5}}-18$$

AVI an anthropometric tool for measuring general volume, was computed from the WC and hip circumference using the following equation:

$$=\frac{[2 cm \times {(WC\left(cm\right))}^{2}+0.7cm\times {(WC(cm)-hip(cm))}^{2}]}{1000}$$

Considering the sexual difference in VAT estimations, VAI seems to be the best approach based on a mathematical model by sex, which indirectly expresses visceral adipose tissue function. This index was calculated based on the values of WC, BMI, TG, and HDL-C by using the following formula:

$$Females: VAI=(\frac{wc (cm)}{36.85+\left(1.89\times BMI(kg/{m}^{2}\right))})\times (\frac{TG (\frac{mmol}{l})}{0.81})\times (\frac{1.52}{HDL (\frac{mmol}{l})})$$

WWI is used to assess adiposity by standardizing WC for weight and was calculated as WC in cm divided by the square root of weight in kg (cm/√kg).

CI was calculated based on the values of WC, weight, and height, by using the following formula:

$$\mathrm{CI }=\frac{WC \left(m\right)}{0.109\sqrt{weight (kg)/height (m)}}-18$$

Lipid accumulation product (LAP) is another adiposity index reflecting lipid accumulation was calculated as the sum of WC and TG.

## Adipose Tissue Samples, RNA Extraction, and Real-Time Quantitative Polymerase Chain Reaction (PCR)

Paired samples of VAT and SAT were obtained during bariatric.surgery in the obese group or during the inguinal hernia or cholecystectomy in the normal-weight subjects as described previously. Following washing with sterile and cold phosphate-buffered saline, the biopsy samples were immediately frozen in liquid nitrogen and kept at 80 °C for further experiments.

Total RNA was isolated from frozen adipose tissue (100 mg) using the RNeasy Lipid Tissue Mini Kit (Qiagen, Germany).

Frozen adipose tissues (100 mg) were transferred into 1 ml QIAzol Lysis Reagent (Qiagen, Hilden, Germany), and homogenized using a pestle and porcelain mortar. Then, total RNA was isolated from the homogenate using the RNeasy Lipid Tissue Mini Kit (Qiagen, Germany). Before reverse transcription, isolated RNAs were checked for quality and quantity with a NanoDrop 2000 Spectrophotometer (Thermo Fisher Scientific) and RNA integrity was checked by running RNA samples on a 1% agarose gel. Complementary DNA (cDNA) was synthesized.from RNA (1 µg) using the PrimeScript 1st Strand cDNA Synthesis kit (Takara, Japan).

Before further gene expression studies, the standard curve was generated for each primer set using a serial dilution of cDNA synthesized from a pool of RNA extracted from adipose tissues (VAT and SAT). Accordingly, the amplification efficiency for each primer was between 90–100%.

The relative mRNA expression was assessed by real-time PCR using BioFACT™ 2X Real-Time PCR Master Mix (For SYBR Green I) in a Step-One-Plus TM real-time (ABI Applied Biosystems). The sequences of gene-specific primers for the target genes; PPARγ, C/EBPα, LXR, SREBP1c, FAS, and ACC, and the reference gene; β-actin are shown in supplementary Table 1. PCR was performed with 15 min of initial denaturation at 95 °C and then followed by 40 cycles with denaturation at 95 °C for 20 s, and annealing at 60 °C for 15 s. It should be noted that each expression was quantified in duplicate. A melting curve analysis was performed to confirm the specificity of all amplified products.

For each sample, the difference in Ct values (ΔCt) between the target gene and the reference gene was calculated. Since the efficiency (E) of amplification was from 90 to 100% in all assays, we used a 2^−∆Ct^ method to perform relative quantification.

## Statistical analysis

Data normality was checked by the Shapiro–Wilk test. Laboratory and anthropometric parameters with normal distribution were presented as mean ± standard deviation (SD), and variables without normal distribution were presented as median (interquartile ranges). All values in the figures were shown as the mean ± standard error of the mean (SEM). Log-transformation was employed for variables with non-normal distribution. The comparison of gene expression levels, as well as anthropometric and biochemical data between obese patients and normal-weight subjects was carried out by independent students t-test on log-transformed variables.

Subsequently, ANCOVA analysis was performed to remove the effects of potential confounders. Correlation coefficients were calculated using the two-tailed Pearson’s correlation analysis. It should be noted that non-normally distributed variables were log-transformed to generate a normal distribution before further analyses. Principal component analysis (PCA) with a varimax rotation was performed to reduce the adipogenic and lipogenic genes into a smaller set of principal components that account for most of the observed variations. It should be noted that the patterns were normalized using the Kaiser method to have greater interpretability. Using PCA results, two significant principal components for adipogenic and lipogenic genes in SAT (SPC1 and SPC2) and VAT (VPC1 and VPC2) were identified as shown in supplementary Table 2 and supplementary Table 3, respectively.

All statistical assessments were two-tailed and P-value < 0.05 was considered statistically significant. All data analysis was performed using SPSS 20 (SPSS, Chicago, IL, USA).

## Results

### Study population characteristics

Some parts of the results regarding the anthropometric, clinical, and metabolic characterizations of the study population which are presented here are related to those in earlier work (unpublished data). The anthropometric and metabolic variables of obese and normal-weight groups are displayed in Table 1. There is no statistically significant difference in terms of age, SBP, DBP, and circulating levels of FBG, urea, HDL-C, TG, AST, and ALT between the two studied groups. However, the circulating levels of LDL-C, total cholesterol, uric acid, creatinine, albumin, total protein, hs-CRP, insulin, HbA1c, and HOMA-IR value were significantly higher in the obese group compared to normal-weight subjects.

**Table 1:** The anthropometric, clinical, and metabolic characterizations of all participants

	Women with normal-weight (*n* = 22)	Women with obesity (*n* = 24)	Total difference *p*-value
Age, years	37.68 ± 9.07	34.91 ± 6.61	0.241
BMI, Kg/m^2^	23.30 (22.84–24.34)	42.59(36.38–46.10)	0.000
VAI,-	1.75 (1.27–2.20)	2.27 (1.34–3.03)	0.132
LAP,-	25.87 ± 13.12	76.69 ± 36.86	0.000
WC(cm)	85 (83–87)	114(111.5–119)	0.000
HC (cm)	95(90–97)	128 (120–133)	0.000
WHR	0.88 ± 0.05	0.91 ± 0.057	0.088
BAI, -	27.81 ± 3.22	43.89 ± 6.79	0.000
WWI,-	10.66 ± 0.7	11.08 ± 0.84	0.073
CI,-	0.89 ± 0.09	1.09 ± 0.11	0.000
AVI,-	14.54 (13.88–15.18)	26.22(25.16–28.59)	0.000
WHtR,-	0.51 ± 0.04	0.72 ± 0.058	0.000
SBP, mmHg	116.09 ± 11.44	121.04 ± 14.52	0.228
DBP, mmHg	75.23 ± 7.63	77.62 ± 12.53	0.568
FBG, mg/dL	85.21 ± 7.28	88.99 ± 8.71	0.119
Fasting Insulin, µU/mL	8.02 ± 3.57	19.42 ± 4.69	0.000
HOMA-IR, -	1.68 ± 0.75	4.25 ± 1.07	0.000
Urea, mg/dL	22.60 ± 7.54	26.53 ± 6.07	0.057
Creatinine, mg/dL	0.58 ± 0.15	0.73 ± 0.11	0.001
Uric acid, mg/dL	4.00 ± 0.83	5.51 ± 1.06	0.000
AST, U/L	16.7(11.7–20.95)	20.65 (16.2–23.95)	0.170
ALT, U/L	12.55 (11.07–20.25)	20.30 (15.35–30.20)	0.209
HDL-C, mg/dL	44.02 ± 7.33	44.95 ± 7.29	0.667
LDL-C,mg/dL	88.69 ± 28.93	113.41 ± 19.65	0.001
TC, mg/dL	147.41 ± 37.60	180.41 ± 25.48	0.001
TG, mg/dL	91.55 (69.70–127.70)	105.95 (67.6–145.3)	0.960
hs-CRP, mg/L	1.8(1.01–2.5)	5.69 (3–11.02)	0.000

Continuous variables with normal and non-normal distribution were described as the mean ± SD and median (IQR), respectively

BMI, body mass index; VAI, visceral adiposity index; BAI, body adiposity index; VI, abdominal volume index; WWI, weight-adjusted waist index; CI; conicity index; WC, waist circumference; HC, hip circumference; WHR, waist-to-hip ratio; WHtR, Waist to Height Ratio; SBP, systolic blood pressure; DBP, diastolic blood pressure; FBG, fasting blood glucose; TG, triglycerides; TC, total cholesterol; HDL-C, high-density lipoprotein cholesterol; LDL-C, low-density lipoprotein cholesterol; AST, aspartate aminotransferase; ALT, alanine aminotransferase; hs-CRP, high-sensitivity C-reactive Protein; AMH, anti-mullerian hormone; HOMA-IR, homeostasis model assessment of insulin resistance; HbA1c, hemoglobin A1C

As for adiposity indices, the anthropometric measurements, including BMI, WC, hip circumference, BAI, AVI, CI, and WHtR were significantly higher in the obese group in comparison with the non-obese group.

Notably, remaining obesity indices including WHR (*p* = 0.088) and WWI (*p* = 0.073) showed an increasing trend albeit with a borderline statistical significance in women with obesity compared to ones with normal weight.

## The mRNA Expression of PPARγ, C/EBPα, LXR, SREBP1c, FAS, and ACC in SAT and VAT from Obese and Normal-Weight Individuals

**Fig. 1:**
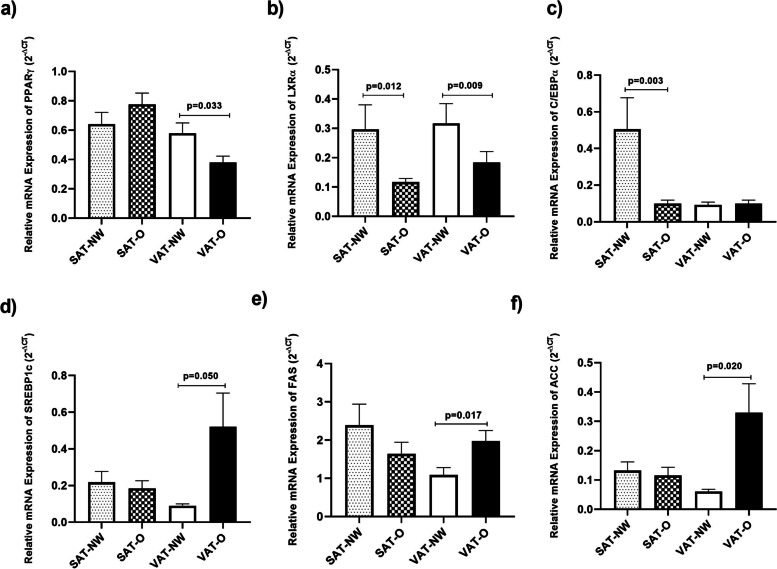
Transcript levels of PPARγ (**a**), LXR (**b**),C/EBPα (**c**), SREBP1c (**d**), FAS (**e**), and ACC (**b**) genes in the visceral (VAT) and subcutaneous (SAT) adipose tissues of obese (O) subjects (*n* = 24) and normal-weight (N) individuals (*n* = 22). Results were shown as mean ± standard error of mean (SEM)

The results of the mRNA expression of PPARγ, C/EBPα, LXR, SREBP1c, FAS, and ACC in SAT and VAT from the two studied groups are shown in Fig. 1.

The mRNA expression of PPARγ was significantly lower in VAT (*p* = 0.033) of obese women compared to normal-weight subjects (Fig. 1a). Moreover, we found that the mRNA expression of LXRα was significantly lower in both VAT (*p* = 0.009) and SAT (*p* = 0.012) from the obese group compared to the non-obese group (Fig. 1b). Although the expression of C/EBPα in the VAT (*p* = 0.75) was not significantly different between the two studied groups, it was lower in SAT (*p* = 0.003) in obese subjects compared to the non-obese group (Fig. 1c).

Conversely, our results revealed higher transcript levels of SREBP1c and its target genes; FAS and ACC in the VAT from obese subjects compared to normal-weight ones (*p* = 0.050, *p* = 0.017, and *p* = 0.02, respectively), whereas the mRNA expression of these genes in the SAT was not significantly different between the two groups (*p* = 0.725 for SREBP1c, *p* = 0.598 for FAS, and *p* = 0.742 for ACC) (Fig. 1d-e). Therefore, it is likely that the expression of PPARγ, C/EBPα, and LXR was lower while the expression of SREBP1c, FAS, and ACC was higher in the obese group compared to the non-obese counterparts.

ANCOVA was performed to remove the possible effect of age and HOMA-IR on the gene expression of all studied genes in VAT and SAT. The results indicated that the decrease in the expression of LXRα both in VAT (*p* = 0.004) and SAT (*p* = 0.002) of the obese group was independent of age and HOMA-IR. Moreover, the C/EBPα transcript level (*P* = 0.02) in the SAT of the obese group remained significantly different from that of the normal-weight group after adjustment for age and HOMA-IR. However, the difference in PPARγ (*P* = 0.131), SREBP1c (*P* = 0.46), FAS (*P* = 0.24), and ACC (*P* = 0.14) in VAT did not remain statistically significant after correction for age and HOMA-IR.

### Univariate Correlations of mRNA Expression of All Studied Genes with Adiposity Indices and Metabolic Profile

We also assessed the Pearson correlation coefficient of PPARγ, C/EBPα, LXR, SREBP1c, FAS, and ACC mRNA levels with adiposity indices and metabolic profiles in SAT (Table 2) and VAT (Table 3) of the whole population.

**Table 2:** Pearson correlation of PPARγ, C/EBPα, LXR, SREBP1c, FAS, and ACC mRNA levels in subcutaneous adipose tissue of whole participants with adiposity indices and metabolic profile

		**mRNA expression in subcutaneous adipose tissue**					
		**PPARγ**	**LXRα**	**CEBPα**	**SREBP1c**	**FAS**	**ACC**
**BMI,Kg/m**^**2**^	**Pearson Correlation**	.182	-.330	-.410	.119	-.023	.034
	***p*-value**	ns	.025	.005	ns	ns	ns
**WC,cm**	Pearson Correlation	.184	-.413	-.410	.127	-.045	.045
	***p*-value**	ns	.004	.005	ns	ns	ns
**HC,cm**	Pearson Correlation	.238	-.376	-.462	.064	-.096	.090
	***p*-value**	ns	.010	.001	ns	ns	ns
**WHR,-**	Pearson Correlation	-.105	-.237	.028	.216	.128	-.109
	***p*-value**	ns	ns	ns	. ns	ns	ns
**WHtR,-**	**Pearson Correlation**	.195	-.425	-.467	.120	-.030	.085
	***p*-value**	. ns	.003	.001	ns	ns	ns
**BAI,-**	**Pearson Correlation**	.240	-.380	-.544	.066	-.059	.145
	***p*-value**	ns	.009	.000	ns	ns	ns
**VAI,-**	**Pearson Correlation**	.003	-.141	-.089	-.019	.005	.067
	***p*-value**	ns	ns	ns	ns	ns	ns
**LAP,-**	**Pearson Correlation**	.110	-.354	-.345	.116	-.021	.062
	***p*-value**	ns	.016	.019	ns	ns	ns
**AVI,-**	**Pearson Correlation**	.186	-.413	-.412	.124	-.048	.048
	***p*-value**	ns	.004	.004	ns	ns	ns
**WWI,-**	**Pearson Correlation**	.107	-.374	-.317	.089	-.008	.137
	***p*-value**	ns	.010	.032	ns	ns	ns
**CI,-**	**Pearson Correlation**	.170	-.457	-.421	.113	-.037	.101
	***p*-value**	ns	.001	.004	ns	ns	ns
**HOMA-IR,**	**Pearson Correlation**	.190	-.118	-.373	.157	-.032	.128
	***p*-value**	ns	ns	.011	ns	ns	ns
**Insulin, µU/mL**	**Pearson Correlation**	.365	-.029	-.259	.229	.070	.169
	***p*-value**	.013	ns	ns	ns	ns	ns

BMI, body mass index; VAI, visceral adiposity index; BAI, body adiposity index; VI, abdominal volume index; WWI, weight-adjusted waist index; CI; conicity index; WC, waist circumference; HC, hip circumference; WHR, waist-to-hip ratio; WHtR, Waist to Height Ratio; HOMA-IR, homeostasis model assessment of insulin resistance; PPARγ: peroxisome proliferator activator receptor γ; C/EBPα: CCAAT/enhancer-binding protein alpha; LXRα: liver X receptor; SREBP1c: sterol regulatory element-binding protein-1c;FAS:fatty acid synthase;ACC:acetyl CoA carboxylase;ns,non-significant

**Table 3:** Pearson correlation of PPARγ, C/EBPα, LXR, SREBP1c, FAS, and ACC mRNA levels in visceral adipose tissue of whole participants with adiposity indices and metabolic profile

		**mRNA expression in visceral adipose tissue**					
		**PPARγ**	**LXRα**	**CEBPα**	**SREBP1c**	**FAS**	**ACC**
**BMI,Kg/m**^**2**^	**Pearson Correlation**	-.299	-.282	-.011	.325	.387	.344
	***p*-value**	.044	ns	ns	.027	.008	.019
**WC,cm**	**Pearson Correlation**	-.251	-.230	-.067	.346	.496	.286
	***p*-value**	ns	ns	ns	.019	.000	ns
**HC,cm**	**Pearson Correlation**	-.368	-.243	-.133	.384	.435	.281
	***p*-value**	.012	ns	ns	.008	.002	ns
**WHR,-**	**Pearson Correlation**	.250	-.040	.177	.000	.322	.102
	***p*-value**	ns	ns	ns	ns	.029	ns
**WHtR,-**	**Pearson Correlation**	-.274	-.297	-.053	.328	.438	.223
	***p*-value**	ns	.045	ns	.026	.002	ns
**BAI,-**	**Pearson Correlation**	-.361	-.329	-.089	.333	.350	.199
	***p*-value**	.014	.026	ns	.024	.017	ns
**VAI,-**	**Pearson Correlation**	-.355	-.242	-.336	.093	.156	-.014
	***p*-value**	.015	ns	.023	ns	ns	ns
**LAP,-**	**Pearson Correlation**	-.367	-.288	-.186	.258	.424	.228
	***p*-value**	.012	.043	ns	ns	.003	ns
**AVI,-**	**Pearson Correlation**	-.258	-.232	-.069	.349	.494	.287
	***p*-value**	ns	ns	ns	.017	.000	ns
**WWI,-**	**Pearson Correlation**	-.019	-.132	-.084	.134	.338	-.123
	***p*-value**	ns	ns	ns	ns	.022	ns
**CI,-**	**Pearson Correlation**	-.186	-.225	-.083	.293	.473	.111
	***p*-value**	ns	ns	ns	.048	.001	ns
**HOMA-IR,**	**Pearson Correlation**	-.260	-.096	-.074	.349	.305	.279
	***p*-value**	ns	ns	ns	.017	.039	ns
**Insulin,µU/mL**	**Pearson Correlation**	-.281	-.185	.017	.275	.222	.296
	***p*-value**	ns	ns	ns	ns	ns	.046

*BMI *Body mass index, *VAI * Visceral adiposity index, *BAI *Body adiposity index, *VI *Abdominal volume index, *WWI *Weight-adjusted waist index, *CI *Conicity index, *WC *Waist circumference,  *HC *Hip circumference, *WHR *Waist-to-hip ratio, *WHtR *Waist to Height Ratio, *HOMA-IR *Homeostasis model assessment of insulin resistance, *PPARγ *Peroxisome proliferator activator receptor γ, *C/EBPα *CCAAT/enhancer-binding protein alpha, *LXRα *liver X receptor, *SREBP1c *Sterol regulatory element-binding protein-1c, *FAS *Fatty acid synthase, *ACC *Acetyl CoA carboxylase,  *ns *Non-significant

Our results indicated that the mRNA expression of PPARγ in the SAT is positively correlated with insulin levels (r = 0.365, *p* = 0.013).

Moreover, SAT mRNA expression of LXR showed an inverse correlation with adiposity indices including BMI (r = -0.330, *p* = 0.025), WC (r = -0.413, *p* = 0.004), HC (r = -0.376, *p* = 0.010), BAI (r = -0.380, *p* = 0.009), CI (r = -0.457, *p* = 0.001), AVI (r = -0.413, *p* = 0.004), WHtR (r = -0.467, *p* = 0.001), WWI (r = -0.317, *p* = 0.032), and LAP (r = -0.354, *p* = 0.016).

Similarly, SAT mRNA expression of C/EBPα was inversely correlated with BMI (r = -0.410, *p* = 0.005), WC (r = -0.410,p = 0.005), HC (r = -0.462, *p* = 0.001), BAI (r = -0.544, *p* = 0.0001), CI (r = -0.421, *p* = 0.004), AVI (r = -0.412, *p* = 0.004), WHtR (r = -0.425, *p* = 0.003), WWI (r = -0.374, *p* = 0.010), and LAP (r = -0.345, *p* = 0.019), and HOMA-IR (r = -0.373, *p* = 0.011).

In this study, according to correlation analysis, mRNA expression of PPARγ in VAT showed a significant inverse relationship with the BMI (r = -0.299, *p* = 0.044), VAI (r = -0.355, *p* = 0.015), LAP (r = -0.367, *p* = 0.012), HC (r = -0.368, *p* = 0.012), and BAI (r = -0.361, *p* = 0.014) in the whole study population. Our results also revealed a significant inverse correlation of VAT LXRα transcript levels with LAP (r = -0.288, *p* = 0.043), BAI (r = -0.329, *p* = 0.026), and WHtR (r = -0.297, *p* = 0.045). Moreover, there was an inverse correlation between VAT C/EBPα transcript levels and VAI (r = -0.336, *p* = 0.023).

On the other hand, VAT SREBP1c mRNA expression showed a significant positive correlation with BMI (r = 0.325, *p* = 0.027), WC (r = 0.346, *p* = 0.019), HC (r = 0.384, *p* = 0.008), WHtR (r = 0.328, *p* = 0.026), BAI (r = 0.333, *p* = 0.024), AVI (r = 0.349, *p* = 0.017), CI (r = 0.293, *p* = 0.048), and HOMA-IR (r = 0.349, *p* = 0.017).

FAS gene expression in the VAT also correlated positively with LAP (r = 0.424, *p* = 0.003), BMI (r = 0.387, *p* = 0.008), WC (r = 0.496, *p* = 0.0001), HC (r = 0.435, *p* = 0.002), WHR (r = 0.322, *p* = 0.029), BAI (r = 0.350, p = 0.017),CI(r = 0.473, *p* = 0.001), AVI (r = 0.494, *p* = 0.0001), WHtR (r = 0.438, *p* = 0.002), and HOMA-IR (r = 0.305, *p* = 0.039). We also found a positive correlation between ACC gene expression in VAT and BMI (r = 0.344, *p* = 0.019), and insulin levels (r = 0.296, *p* = 0.046).

Altogether, it seems that the expression level of SREBP1c, FAS, and ACC had a positive correlation with obesity indices as well as HOMA-IR while the expression level of PPARγ, C/EBPα, and LXR had an inverse correlation with adiposity indices in all subjects.

### Correlation Analysis of the Transcript Level Patterns of Adipogenic and Lipogenic Genesin SAT and VAT of Whole Participants with Adiposity Indices and Metabolic Profile

Using PCA results, we identified two significant principal components (SPC1 and SPC2) for adipogenic and lipogenic genes in SAT. SPC1 was characterized by relatively high transcript levels of SREBP1c, PPARγ, FAS, and ACC. However, the second pattern (SPC2) was associated with C/EBPα and LXR α mRNA expression.

Similarly, two patterns for adipogenic and lipogenic genes were identified (VPC1 and VPC2) in VAT. Specifically, the first pattern was characterized by transcript levels of SREBP1c, FAS, and ACC. The second one was characterized by C/EBPα, LXR α, and PPARγ mRNA expression.

Next, Pearson’s correlation analysis was used to assess the possible association of each lipogenic and adipogenic pattern score with obesity indices and insulin resistance indices (Table 4).

**Table 4:** Pearson correlation of pattern of the transcript level of adipogenic and lipogenic genes in subcutaneous and visceral adipose tissue of whole participants with adiposity indices and metabolic profile

		**SPC1**	**SPC2**	**VPC1**	**VPC1**
**BMI,Kg/m**^**2**^	**Pearson Correlation**	.160	-.492	.398	-.222
	***p*-value**	ns	.001	.006	ns
**WC,cm**	**Pearson Correlation**	.154	-.530	.433	-.178
	***p*-value**	ns	.000	.003	ns
**HC,cm**	**Pearson Correlation**	.158	-.549	.440	-.281
	***p*-value**	ns	.000	.002	.059
**WHR,-**	**Pearson Correlation**	.032	-.104	.113	.237
	***p*-value**	ns	ns	ns	ns
**WHtR,-**	**Pearson Correlation**	.182	-.579	.369	-.219
	***p*-value**	ns	.000	.012	ns
**BAI,-**	**Pearson Correlation**	.025	-.129	.118	-.380
	***p*-value**	ns	ns	ns	.009
**VAI,-**	**Pearson Correlation**	.131	-.445	.355	-.311
	***p*-value**	ns	.002	.015	.035
**LAP,-**	**Pearson Correlation**	.203	-.615	.335	-.305
	***p*-value**	ns	.000	.023	.039
**AVI,-**	**Pearson Correlation**	.140	-.429	.120	-.035
	***p*-value**	ns	.003	ns	ns
**WWI,-**	**Pearson Correlation**	.163	-.554	.329	-.145
	***p*-value**	ns	.000	.026	ns
**CI,-**	**Pearson Correlation**	.154	-.531	.434	-.183
	***p*-value**	ns	.000	.003	ns
**HOMA-IR,**	**Pearson Correlation**	.202	-.374	.387	-.168
	***p*-value**	ns	.011	.008	ns
**Fasting Insulin, µU/mL**	**Pearson Correlation**	.301	-.280	.306	-.190
	***p*-value**	.042	.060	.038	.206

Four patterns were identified using principal component analysis for adipogenic and lipogenic genes in subcutaneous (SPC1 and SPC2) and visceral (VPC1 and VPC2) adipose tissue.

SPC1 was characterized by high transcript levels of SREBP1c, PPARγ, FAS, and ACC.

SPC2 was associated with C/EBPα and LXR α mRNA expression.

VPC1 was characterized by transcript levels of SREBP1c, FAS, and ACC.

VPC2 was identified by C/EBPα, LXR α, and PPARγ mRNA expression.

BMI, body mass index; VAI, visceral adiposity index; BAI, body adiposity index; VI, abdominal volume index; WWI, weight-adjusted waist index; CI; conicity index; WC, waist circumference; HC, hip circumference; WHR, waist-to-hip ratio; WHtR, Waist to Height Ratio; SBP, systolic blood pressure; DBP, diastolic blood pressure; FBG, fasting blood glucose; TG, triglycerides; TC, total cholesterol; HDL-C, high-density lipoprotein cholesterol; LDL-C, low-density lipoprotein cholesterol; AST, aspartate aminotransferase; ALT, alanine aminotransferase; AMH, anti-mullerian hormone; HOMA-IR, homeostasis model assessment of insulin resistance; HbA1c, hemoglobin A1C; ns,non-significant

As shown in Table 4, SPC1 was positively correlated with insulin level (r = 0.301, *p* = 0.042) in the whole study population. However, SPC2 was inversely correlated with BMI (r = -0.492, *p* = 0.001), WC (r = -0.530,*p* = 0.0001), HC(r = -0.549, *p* = 0.0001), WHtR (r = -0.579, *p* = 0.0001), VAI (r = -0.445, *p* = 0.002), LAP (r = -0.615, *p* = 0.0001), AVI (r =—0.429, *p* = 0.003), WWI (r =—0.554, *p* = 0.0001), CI (r = -0.531, *p* = 0.0001), HOMA-.IR (r = -0.374, *p* = 0.011) in the whole study population.

The VPC1 was significantly correlated with BMI (r = 0.398, *p* = 0.006), WC (r = 0.433,*p* = 0.003), HC(r = 0.440, *p* = 0.002), WHtR (r = 0.396, *p* = 0.012), VAI (r = 0.355, *p* = 0.015), LAP (r = 0.3035, *p* = 0.023), WWI (r = 0.329, p = 0.026), CI (r = 0.434, *p* = 0.003), insulin level (r = 0.387, *p* = 0.008), and HOMA-IR (r = 0.306, p = 0.038) in the whole study population. However, BAI (r = -0.380, *p* = 0.009), VAI (r = -0.311, *p* = 0.035), and LAP (r = -0.305, *p* = 0.039) showed an inverse correlation with VPC2.

Remarkably, the observed results based on PCA data was almost in parallel to individual expression levels of adipogenic and lipogenic factors and their correlation with inulin value and adiposity indices. Indeed, the association of the identified patterns, especially SPC2 and VPC1 with adiposity parameters as well as insulin resistance demonstrated significant p-values.

## Discussion

The alteration in the gene expression of lipogenic and adipogenic factors in relation with obesity and associated metabolic abnormalities has been reported in numerous pieces of evidence although with inconsistent results. Moreover, this association has been mostly investigated individually [27, 28]. Therefore, the present study aimed to investigate the association of the gene expression of lipogenic and adipogenic factors pattern with insulin resistance and a variety of anthropometric indices in the context of obesity in humans. In particular, here, we explain it.from two perspectives; (i) individually and [2] PCA-derived findings.

According to the first perspective, we found a reduced transcript level of PPARγ, C/EBPα, and LXRwhile the expression level of SREBP1c, FAS, and ACC was higher in adipose depots of subjects with obesity compared to the non-obese counterparts. This association was further substantiated by correlation analysis, as revealed that transcript levels of SREBP1c, FAS, and ACC had a positive correlation with a variety of adiposity indices and HOMA-IR values. However, the transcript level of PPARγ, C/EBPα, and LXR displayed an inverse correlation with the indicated parameters. Of great interest, the involvement of adipogenic and lipogenic has been reported in the context of obesity, albeit with contrary results. For instance, some studies reported a higher expression of PPARγ and SREBP1c in obese subjects [29, 30], while others observed the opposite results in this regard [15, 31]. Also, a limited number of data showed a dysregulated expression of LXRα in the context of obesity in humans [14]. In contrast to our findings, Ranganathan G et al*.* reported the down-regulation of FAS in adipose tissues of subjects with impaired glucose tolerance [19]. Nevertheless, another study reported the up-regulation of FAS in adipose tissue of obese subjects which was linked to impaired insulin sensitivity and visceral fat accumulation [20].

According to the second perspective based PCA-derived data, two separate patterns for adipogenic and lipogenic genes in SAT and VAT were obtained. In SAT, the first pattern was characterized by relatively high transcript levels of SREBP1c, PPARγ, FAS, and ACC. However, the second pattern was associated with C/EBPα and LXR α mRNA expression. In VAT, one pattern included transcript levels of SREBP1c, FAS, and ACC while, the second one was linked to C/EBPα, LXR α, and PPARγ mRNA expression. Interestingly, the link between the identified patterns, especially the second pattern in SAT and the first pattern in VAT and adiposity parameters, and insulin resistance yielded significant *p*-values.

To further explain the second perspective, it is crucial to mention that adipose tissue dysfunction is a principal contributor to obesity and related metabolic abnormalities which primarily results from impaired transcriptional regulation of the key factors that control lipogenesis and adipogenesis. A complex and finely well-organized transcriptional network (cascade) controls adipogenesis and lipogenesis. Hence, it seems that the evaluation of a combination of adipogenic and lipogenic factors could, at least partly, clarify obscure issues in the pathogenesis of obesity [27]. In this way, PCA is not only used as a powerful method to recognize the clustering of adipogenic and lipogenic genes in the context of human obesity, but is also applied to the data reduction of the existing 6 orthogonal variables. Subsequently, linear regression was performed to provide detailed information about quantitative associations between the PCA-obtained pattern and adiposity indices.

Induction of adipocyte differentiation is mediated by a combination of adipogenic transcription factors; PPARγ, CEBPα, and LXRα, and the subsequent lipid storage by a lipogenic transcription factor; SREBP1c [29, 32]. Adipogenesis is the process of pre-adipocyte differentiation to lipid-laden adipocytes and has a central role in systemic energy homeostasis. However, lipogenesis is a process that happens preferentially in adipose tissue and encompasses the synthesis of fatty acids and triglycerides as energy reserves. A well-functioning adipose tissue can sequester nutritional overload in the form of triglycerides and therefore have a protective role against detrimental effects of lipid storage in other organs [8]. The expression of adipogenic genes usually shows that how the adipose tissue expands [33]. In line with this, a decreased expression of PPARγ, CEBPα, and LXRα in adipose tissue of women with obesity can reflect low adipose tissue expansion in the present study. Remarkably, previous evidence points out that C/EBPα and PPARγ are principal players in adipogenesis and participate in a single pathway of adipocyte formation with PPARγ being the master regulator of adipogenesis [34]. Remarkably, the failure of fat accumulation was reported before in CEBPα knockout mice immediately after birth. Moreover, male PPARγ knockout mice were more insulin resistant on a normal diet in comparison with the wild-type ones. Indeed, this study indicated that impaired adipogenesis could trigger metabolic abnormalities, such as increased adiposity markers and insulin resistance in obese cases [35]. It was also suggested that adipose tissue-specific ablation of PPARγ causes progressive lipodystrophy [36]. Intriguingly, VAI and LAP have been proposed as substitute indicators of dysfunction and distribution of adipose tissue that indirectly predict cardiometabolic risk [37, 38]. Here, the SPC2 pattern score characterized by C/EBPα and LXRα expression was also associated with VAI and LAP expressing lipid accumulation and adipose tissue dysfunction. It is important to note that.an impaired fat deposition in adipose tissue is closely linked to lipotoxicity and subsequently lipid accumulation in the liver, skeletal muscle, and pancreas, which in turn lead to the development of insulin resistance and other abnormalities pertinent to obesity [39].

This concept was strengthened by increased expression of lipogenic genes; SREBP1c, FAS, and ACC too. Indeed,

SREBP1c which promotes lipogenesis by inducing lipogenic enzymes; FAS and ACC and in turn, leads to increased lipid accumulation within the adipocyte [40] was reported to be overexpressed in obese subjects compared to the non-obese ones. To support this notion, VPC1 reflecting high transcript levels of SREBP1c, FAS, and ACC was significantly correlated with LAP and VAI as well.

Here, we identified several potential patterns of adipogenic and lipogenic genes in adipose tissues using PCA for future investigations. However, it should be noted that this research area needs a more comprehensive study considering other factors in adipose tissue function. Also, it should be noted that the measurement of adiponectin transcript level, as a major component of adiposity can provide valuable information on in-depth interpretation of our results. Finally, we would like to stress that further studies on both sexes and with a larger sample size are required to strengthen the results.

## Conclusion

The present study used PCA to simultaneously evaluate the expression levels of the key genes involved in the complex transcriptional network regulating adipogenesis and lipogenesis; comprising of PPARγ, C/EBPα, LXR, SREBP1c, FAS, and ACC in SAT and VAT from obese and normal-weight subjects to decrease the adipogenic and lipogenic genes into a smaller set of principal components that account for most of the observed variations. Interestingly, this study provided valuable information on multiple patterns for adipogenic and lipogenic genes in adipose tissues with insulin resistance and a variety of anthropometric indices predicting adipose tissue dysfunction and lipid accumulation in the context of obesity.

## Supplementary Information

**Additional file 1: Table S1.** Forward and reverse primers used for real-time PCR. **Table S2.** Principal factor loading of transcript levels of adipogenic and lipogenic genes **Table S3.** Principal factor loading of transcript levels of adipogenic and lipogenic genes.

## Data Availability

The datasets used and/or analyzed during the current study are available from the corresponding author on reasonable request.
