# Supplementary material for: Principal component analysis of adipose tissue gene expression of lipogenic and adipogenic factors in obesity
Source: BMC Endocr Disord. 2023 Apr 27;23:94. doi: 10.1186/s12902-023-01347-w (PMC10134674; doi:10.1186/s12902-023-01347-w)
Supplement: Supplementary file 1 — Additional file 1: Table S1. Forward and reverse primers used for real-time PCR. Table S2. Principal factor loading of transcript levels of adipogenic and lipogenic genes Table S3. Principal factor loading of transcript levels of adipogenic and lipogenic genes. [file 12902_2023_1347_MOESM1_ESM.docx]

| **Table S1: Forward and reverse primers used for real-time PCR.** | | |
| --- | --- | --- |
| Primer | Forward sequence | Reverse sequence |
| PPARγ | 5'-GAGTACCAAAGTGCAATCAAAG-3' | 5'-CTCCGGAAGAAACCCTTGCATC-3' |
| C/EBP-α | 5'- CGAGTCACACCAGAAAGCTAGG -3' | 5'- CTTGTCATAACTCCGGTCCCTC -3' |
| LXRα | 5'- CGCACTACATCTGCCACAGT -3' | 5'- GTTCTTCTGACAGGACACACTCC -3' |
| SREBP-1c | 5'-GGATGGTGTTCACTCGGTA-3' | 5'-GGTGATATGTGTCTGCGTC-3' |
| FAS | 5'-GAGGAAGGAGGGTGTGTTT-3' | 5'-CGGGGATAGAGGTGCTGA-3' |
| ACC | 5'-TGAGGACAGCAAGGCAAG-3' | 5'-CAGGACAGGCAGAGGAAGA-3' |
| β-actin | 5'-TCCTTCCTGGGCATGGAGT-3' | 5'-ACTGTGTTGGCGTACAGGTC-3' |
| PPARγ, peroxisome proliferator-activated receptor gamma; C/EBP-α ,CCAAT/enhancer-binding protein alpha ;LXR,liver X receptor ;SREBP-1c, sterol regulatory element-binding protein 1c; FAS, fatty acid synthase; ACC, acetyl-CoA carboxylase. | | |

| **Table S2: Principal factor loading of transcript levels of adipogenic and lipogenic genes** | | |
| --- | --- | --- |
|  | Component | |
|  | SP1 | SP2 |
| FAS | .862 | .179 |
| SREBP1c | .835 |  |
| ACC | .775 | .218 |
| PPARγ | .705 |  |
| C/EBPα |  | .936 |
| LXRα | .502 | .694 |
| Percent of variance explained | 46.499 | 24.095 |
| Extraction Method: Principal Component Analysis.  Rotation Method: Varimax with Kaiser Normalization. | | |
| 1. Rotation converged in 3 iterations.   PPARγ: peroxisome proliferator activator receptor γ; C/EBPα: CCAAT/enhancer-binding protein alpha; LXRα: liver X receptor ; SREBP-1c: sterol regulatory element-binding protein-1c ;FAS:fatty acid synthase;ACC:acetyl CoA carboxylase | | |

| **Table S3: Principal factor loading of transcript levels of adipogenic and lipogenic genes** | | |
| --- | --- | --- |
|  | Component | |
|  | VP1 | VP2 |
| SREBP1c | .862 | -.107 |
| ACC | .755 |  |
| FAS | .552 | .337 |
| PPARγ |  | .799 |
| LXRα | .193 | .672 |
| C/EBPα | -.304 | .655 |
| Percent of variance explained | 29.171 | 27.446 |
| Extraction Method: Principal Component Analysis.  Rotation Method: Varimax with Kaiser Normalization. | | |
| 1. Rotation converged in 3 iterations.   PPARγ: peroxisome proliferator activator receptor γ; C/EBPα: CCAAT/enhancer-binding protein alpha; LXRα: liver X receptor ; SREBP-1c: sterol regulatory element-binding protein-1c ;FAS:fatty acid synthase;ACC:acetyl CoA carboxylase | | |
